# Environmental impact and nutrient adequacy of derived dietary patterns in Vietnam

**DOI:** 10.3389/fnut.2023.986241

**Published:** 2023-07-06

**Authors:** Son D. Nguyen, Sander Biesbroek, Tuyen D. Le, Edith J. M. Feskens, Inge D. Brouwer, Elise F. Talsma

**Affiliations:** ^1^Division of Human Nutrition and Health, Wageningen University and Research, Wageningen, Netherlands; ^2^National Institute of Nutrition, Ministry of Health, Hanoi, Vietnam; ^3^International Food Policy Research Institute, Washington DC, United States

**Keywords:** nutrition survey, diet quality, greenhouse gas emission, blue water use, principal component analysis

## Abstract

**Rationale and objective:**

Improving diet quality while decreasing environmental impacts is an important challenge for a healthy and sustainable food system. Therefore, this study aims to analyze the most common dietary patterns per female household member and explore the diet quality and environmental impacts of these patterns.

**Methodology:**

The nationally representative General Nutrition Survey of 2009–2010 (*n* = 8,225 households) was used to derive dietary patterns using principal component analysis (PCA) based on 18 food groups as input variables. Quintiles of the highest adherence (Q5) and lowest adherence (Q1) were generated based on the factor score of each dietary pattern. Nutrient adequacy and dietary diversity scores (DDS) were calculated to measure diet quality, and greenhouse gas emission (GHGE) and blue water use (BWU) were selected as environmental impact indicators.

**Results:**

Using PCA, three distinct dietary patterns were identified: an Omnivorous, Traditional, and Pescatarian pattern. Compared to the Traditional pattern, the Omnivorous and Pescatarian patterns (Q5s) were associated with a higher nutrient adequacy, with mean probability of adequacy of 0.51 in both patterns, compared to 0.45 in the Traditional pattern. However, environmental impacts in terms of GHGE and BWU per 2,000 kcal were considerably higher in the Omnivorous pattern (6.14 kg CO_2_-eq. and 0.15 m^3^/kg) compared to all other pattern’s Q5s. The GHGE was lowest in the Traditional pattern (4.18 kg CO_2_-eq.) and the Pescatarian pattern has the lowest BWU (0.12 m^3^/kg).

**Conclusion:**

Despite that diet quality was slightly better in all three patterns compared to the average diet of the total population, environmental impact was also higher. Therefore, future research is needed to develop a more optimal diet that considers both diet quality and environmental impact to explore the trade-offs between diet quality and environmental impact.

## 1. Introduction

Current food systems are challenged by an increasing demand to deliver sufficient and nutritious foods while simultaneously decreasing environmental impact (1, 2). Although it is possible to produce enough food globally to provide every person approximately 2,700 kcal [average requirement energy for men is 2,900 and 2,200 kcal for women (3)], more than 800 million people still suffer from chronic undernutrition (4). In addition, hidden hunger such as iron, vitamin A and zinc deficiencies still exists when many diets with sufficient calories do not provide adequate amounts of micronutrients (5, 6). Aside from health, diet has an impact on the environment through production and transport of foods (7, 8). Food systems contribute up to third of total global anthropogenic greenhouse gas emissions (GHGE) (9) and agriculture is responsible for 80–86% of food systems emissions and 70% of all water use (10, 11). The livestock sector contributes to 5.6–7.5 Gt CO_2_-eq. of human-induced GHGE from 1995 to 2005 (12). A requirement for a healthy and sustainable food system is to provide foods that are not only beneficial to a healthy diet but also limit harmful environmental impacts, i.e., to balance between animal-based sources and plant-based sources.

Although there is no universally agreed set of indicators to measure the quality of diets, it can be estimated by assessing two main dimensions: adequacy of energy and nutrients; and food diversity (13–15). Currently, the probability approach is the most accurate method to assess the adequacy of nutrient intakes (16) by comparing the nutrient requirement and nutrient intake distributions. The probability of inadequate intake would naturally be very low for those with higher nutrient intakes and would be higher for those with lower nutrient intakes. This method requires the mean and shape of the requirement distribution for a particular nutrient which is only obtained from the quantitative dietary intake survey data (16). But this approach needs accurate quantitative dietary intake data which are often not available due to high costs, time demand, and low capacity available for data collection and analysis in low and middle-income countries. Therefore, a more simple dietary diversity score (DDS), validated as a proxy for micronutrient adequacy (15) is often used, not only to assess food diversity but also diet quality.

Following the 1986 “Doi Moi” or economic reform in Vietnam, when the government promoted food production of especially rice and livestock, and moved to a more liberal market (5, 6), the economy developed exponentially (GDP and foreign investment increased). Accordingly, the diets of Vietnamese people began to gradually include a greater proportion of animal products (17–19), with changes happening faster in urban and high-income households than in rural and low-income households (19).

Several studies examined the greenhouse gas emission (GHGE) and blue water use (BWU) (the volume of surface and groundwater was withdrawn to produce the product) related to food production and consumption (20–23). In Vietnam, half of the total GHGE (from all sectors) comes from agriculture, which includes rice farming, raising livestock, emission from arable land, and burning of agricultural products (24). However, high-quality data on this is lacking. Although Heller et al. (25) found that the per capita diet-related GHGE increased in Vietnam over the period 1971–2011, mainly associated with an increase in meat consumption, this was derived using FAO food balance sheets and not data collected with methods of a higher accuracy (e.g., 24-h recall, food frequency questionnaire; FFQ) (25). One study did utilize a higher quality data collection method (namely, 24-h recall), however, they only did so in northern Vietnam, meaning these results are not nationally representative (26). In order to amend this knowledge gap, this paper aimed to use the nationally representative General Nutrition Survey 2009–2010 to identify common dietary patterns in Vietnam, quantify their GHGEs and BWU, and compare their diet quality and environmental impact to average diets to evaluate potential trade-offs between healthy diets and environmental sustainability objectives.

## 2. Materials and methods

### 2.1. Population

In this study, a secondary analysis of the nationally representative General Nutrition Survey (GNS) of Vietnam was performed. The GNS is conducted every 10 years by the National Institute of Nutrition (NIN) and includes data on demographics, wealth index, anthropometrics, and household dietary intake. The methodology and survey population are described in the General Nutrition Survey 2009–2010 report (27). In short, the GNS included 8,267 households and all persons responsible for preparation of food in the household were interviewed in-person to recall the food they purchased and the food consumed by all household members in the preceding 24 h. A photo book was used to determine the portion size in grams of food items, i.e., rice, beef, pork, chicken, and fruits and vegetables. Standardized questionnaires were used to collect vital information (age, sex, regions, etc.) and to quantify the wealth index [a composite measure of a household’s cumulative living standard used in the Demographic and Health Survey (28)].

### 2.2. Dietary intake data

The dataset comprised quantities of food items consumed in raw, non-cooked form. Retention factors were applied to account for nutrient losses through food preparation (29). To estimate the individual intake from household data, the Adult Male Equivalent (AME) approach was introduced where each individuals intake was expressed as a ratio of the energy requirement of that individual against the energy requirement of the male adult (30, 31). As in nutrition, adult women were one of the most vulnerable groups for nutrient deficiencies and intake. Consequently, in this study, we used the Adult Female Equivalent (AFE) concept. The reference AFE was set at 1 and reflected a female person of 20–30 years of age with 59.4 kg weight and with moderate physical activity level (32, 33). All household members were expressed as proportion of AFE based on their energy requirement and these were summed to arrive at total household AFE. Total household intake was divided by total AFE to arrive at average food intake per AFE per household.

The 8 main food groups from the Vietnam food pyramid recommendations were disaggregated into 18 food groups (Supplementary Table S1) based on health aspects (Vietnamese food pyramid) as well as environmental impact (GHGE and BWU). Rice and non-rice starchy staples were separated from the grain’s food group, because of the differences of cultivation methods which can lead to different impact of water use and GHGE (34). For a similar reasons as well as negative health implications the large food group “meat, egg, fish and seafood” was disaggregated into the six small food groups “white meat,” “red meat,” “processed meat,” “organs,” “fish and seafood,” and “eggs.” For the remaining food groups, the differences of GHGE and blue water use were the main reason for disaggregation. The consumption of these food groups was expressed in grams per AFE per day, and these values were used for the subsequent dietary pattern analysis.

### 2.3. Dietary pattern analysis

To determine the dietary patterns, the 18 food groups were used as input variables in a principal component analysis (PCA) by using the PROC FACTOR function in SAS v9.4. Principal components are linear combinations of the input variables that describe variation in the data, with the first principal component describing the largest portion of the variance, and each component after that describing progressively less (35). Each component describes a dietary pattern, and the linear combination allows the calculation of a component score for each individual; the higher the score, the higher the adherence to this pattern. The patterns described by each component can be interpreted by its factor loadings, which are the correlations between the component and each food group input variable. Both the Scree plot and factor Eigenvalues were used to assess the number of patterns to retain. Quintiles of adherence were generated based on the factor score of each dietary pattern.

### 2.4. Diet quality

Mean probability of nutrient adequacy (MPA) (16) and DDS (36) were used to assess the diet quality of the derived diet patterns.

#### 2.4.1. Mean probability of nutrient adequacy

The probability approach was selected to assess the nutrient intake adequacy (16). We selected 11 nutrients (Vitamin A, C, B6, B12, E, zinc, folate, iron, calcium, magnesium, and protein) to calculate the nutrient adequacy (Supplementary Table S2), as these micronutrients are a public health concern in women of reproductive age in Asia (37–40). In Vietnam in particular, vitamin A, zinc, folate, and iron deficiencies are highly prevalent (41), whereas the prevalence of deficiencies of other nutrients is unclear, in part due to low research priority. The estimated average requirement (EAR) and its distribution were used to assess the probability of adequate nutrient intake (PA) (42). In this paper, the EAR was adapted based on the Vietnamese recommended dietary allowance (RDA) 2015. The EARs of vitamin C, B6, B12, and zinc were calculated based on the RDAs of Vietnamese RDA 2015 report. The PROBNORM function in SAS was used to calculate the probability that an individual intake is above the EAR per nutrient (42, 43). The PA of iron was calculated based on the IOM 2006 suggestion on assessment of iron intake (42). Here, we use iron bioavailability of 5% based on the estimate for a simple and monotonous diet based on mainly cereal, as suggested by the recommendation of ILSI for Southeast Asia (44, 45). The probability of calcium adequacy was based on the method described by Foote et al. which compared intake levels to the adequate intake (AI) (1,000 mg) (46). The mean probability of adequate micronutrient intake (MPA) was calculated as the average of all PAs for protein and the 11 nutrients and standardized for 2,000 kcal (46). A MPA cut-off value of 0.5 was used to determine low micronutrient intakes, as suggested by Kennedy et al. (43).

#### 2.4.2. Dietary diversity score

Dietary diversity was based on the minimum DDS for women of reproductive age (MDD-W) (36). All food items were classified into 10 food groups and assigned a score of 1 if the consumption was greater or equal to 15 g and 0 if not, according to the MDD-W (36). With a scale of DDS from 0 to 10, higher scores suggested higher diversity of diet, and a score of 5 or more reflects achieving minimum dietary diversity and low risk of micronutrient inadequacy.

### 2.5. Environmental impact

Greenhouse gas emission (kg CO_2−_eq) and blue water use (m^3^) were selected to explore the environmental impact. Firstly, for the GHGE, we conducted a literature review and compared various available databases. From the food list of the survey, all food items were matched with food items of the Clune dataset (47), and GHGE was calculated using the highest GHGE value of the range given per food in the Clune dataset (47) and expressed per kg food consumed. Blue water use for food was linked to the existing values from a database made publicly available by the Water Footprint Network (WFN) and Springmann et al. (7, 48). However, there is no information about the water use of condiments, alcohol, and sweet foods from these databases, so these food groups were excluded from water use analysis. For each individual, we summed the GHGE and the blue water use from each food to estimate the total environmental impact of the diet. In addition, the diet-related environmental impact was standardized per 2,000 kcal in order to focus on the differences of diet quality rather than quantity.

### 2.6. Statistical analysis

Data were analyzed using SAS v9.4. The final dataset comprised 8,225 individuals with complete dietary data. For the MPA analyses, the PAs (except for Iron and Calcium) were calculated using the following equation (43):


$$PA=PROBNORM\left(\frac{\bar{y}-r}{SD}\right)\;\;$$

where $\bar{y}$ is an estimated observed intake, r is the EAR of the nutrient and SD is the standard deviation of the EAR. The Cohran-Mantel-Haenzel (CMH) test and linear regression were performed to test the homogeneity over all quintiles of the dietary patterns.

## 3. Results

### 3.1. Derived dietary patterns

Using PCA, three dietary patterns were identified (Table 1). The first dietary pattern explained 6.0% of the variation and was characterized by high positive factor loadings for non-rice starchy staples, red meat, organs, dairy products and fruits, and negative loadings for rice and fish and seafoods; it was named the Omnivorous pattern. The second pattern was named the Traditional pattern. This pattern explained 4.6% of the variation and was characterized by high positive loadings for rice, vegetables rich in vitamin A and vitamin C, and fats, and negative loadings for non-rice starchy staples, oils, fish, and seafood. The third dietary pattern, which explained 4.1% of the variation, was distinguished by high loadings for condiments, oil, fish and sea foods, and rice, and was therefore named the Pescatarian pattern.

**Table 1:** Food group factor loadings of the principal component analysis derived dietary patterns^a^ of household (per AFE) in the Vietnam General nutrition survey 2009–2010 dataset.

	Factor 1 Omnivorous pattern	Factor 2 Traditional pattern	Factor 3 Pescatarian pattern
Rice	−38	47	45
Non-rice starchy staples	48	−23	–
Root, tubers, nuts, and seeds	23	35	–
Red meat	37	31	–
White meat	26	–	–
Organs	42	–	–
Processed meat	27	–	–
Fish and seafood	−22	−44	51
Eggs	23	–	–
Milk and dairy products	34	–	–
Rich vitamin vegetables	–	53	–
Other vegetables	22	–	34
Fruits	51	–	23
Oils	–	−32	54
Fats	–	55	–
Condiments	–	–	60
Sweet foods	34	–	–
Liquor and alcohol	22	–	–

^a^Only food groups with factor loading ≥0.2 were displayed, (−) indicated that the food group has factor loading < 0.2. The printed values were multiplied by 100 and rounded to the nearest integer.

Adherence to the Omnivorous and Pescatarian patterns were positively associated with the wealth index, whereas an inverse association was observed with the Traditional pattern (Table 2). The population of the Red River delta adhered most to the Omnivorous pattern, the population from the Northern mountainous region most to the Traditional pattern, and the population from the North Central and Coastal region consumed mostly to the Pescatarian pattern. The Pescatarian pattern was positively associated with fish, oil, and condiments while the Traditional pattern was not. However, the Traditional and Pescatarian patterns were positively associated with the consumption of rice and vegetables; in contrast, the Omnivorous pattern was inversely associated with the consumption of rice, as well as fish and seafood.

**Table 2:** Participant characteristics and dietary intake by three dietary patterns of household (per AFE) in the Vietnam General nutrition survey 2009–2010 dataset (*n* = 8,225).

	Total population	Omnivorous pattern		Traditional pattern		Pescatarian pattern	
		Q1	Q5	Q1	Q5	Q1	Q5
	*n* = 8,225	*n* = 1,645	*n* = 1,645	*n* = 1,645	*n* = 1,645	*n* = 1,645	*n* = 1,645
Wealth index (%)							
Lowest	20	39.0	7.4^ii^	11.4	31.2^ii^	20.6	16.5^ii^
Second	20	26.6	9.5	17.6	24.7	19.8	18.1
Third	20	19.0	15.3	20.3	17.7	19.6	20.0
Fourth	20	10.8	25.2	23.2	15.7	19.0	22.1
Highest	20	4.7	42.6	27.5	10.8	20.9	23.3
Region (%)							
Red river delta	16.9	9.0	25.1^i^	6.9	23.5^ii^	19.9	16.0^ii^
Northern mountainous	24.4	23.8	20.7	3.8	55.1	33.5	13.9
North Central and Coastal	24.6	32.7	15.6	40.9	9.0	20.2	27.4
Central Highland	7.1	10.3	5.5	6.1	5.0	2.6	11.0
Southeast	8.5	3.8	16.1	11.8	2.4	6.3	10.7
Mekong delta	18.5	20.7	17.1	30.6	5.0	17.6	21.0
Food group intake (g/d) (mean, SD)							
Rice	365 (152)	484 (175)	296 (133)^ii^	280 (110)	484 (187)^ii^	257 (105)	461 (192)^ii^
Non-rice starchy staples	36 (73)	4 (14)	93 (124)^ii^	68 (108)	17 (51)^ii^	66 (115)	27 (54)^ii^
Root, tubers, nut and seeds	32 (74)	9 (30)	56 (108)^ii^	8 (26)	78 (125)^ii^	40 (81)	30 (71)^ii^
Red meat	54 (76)	12 (32)	96 (106)^ii^	23 (40)	89 (110)^ii^	54 (70)	58 (82)^n^
White meat	13 (53)	1 (9)	35 (91)^ii^	6 (30)	15 (56)^ii^	17 (59)	14 (53)^n^
Organs	12 (38)	1 (6)	36 (70)^ii^	13 (36)	9 (33)^i^	11 (33)	15 (40)^ii^
Processed meat	2 (10)	0 (1)	6 (20)^ii^	1 (6)	2 (12)^ii^	3 (16)	1 (6)^ii^
Fish and seafood	65 (84)	109 (115)	46 (74)^ii^	136 (118)	26 (52)^ii^	18 (31)	134 (126)^ii^
Eggs	12 (25)	3 (11)	21 (38)^ii^	6 (16)	17 (33)^ii^	9 (20)	14 (27)^ii^
Milk and dairy products	10 (35)	1 (6)	33 (64)^ii^	9 (30)	13 (44)^ii^	5 (17)	22 (56)^ii^
Rich vitamin vegetables	109 (119)	126 (141)	107 (122)^ii^	42 (60)	213 (165)^ii^	84 (97)	135 (149)^ii^
Other vegetables	112 (108)	79 (93)	146 (121)^ii^	99 (91)	139 (136)^ii^	62 (67)	170 (142)^ii^
Fruits	49 (100)	5 (22)	138 (161)^ii^	57 (112)	41 (95)^ii^	22 (52)	92 (149)^ii^
Oils	5 (10)	4 (6)	8 (13)^ii^	11 (16)	2 (6)^ii^	1 (3)	13 (17)^ii^
Fats	3 (8)	4 (11)	2 (6)^ii^	0 (2)	9 (14)^ii^	4 (11)	2 (7)^ii^
Condiments	18 (17)	17 (15)	21 (22)^ii^	16 (13)	22 (24)^ii^	9 (6)	33 (28)^ii^
Sweet foods	5 (25)	1 (4)	17 (48)^ii^	11(36)	3 (15)^ii^	3 (12)	11 (38)^ii^
Alcohol and beverages	8 (43)	2 (11)	23 (85)^ii^	7 (43)	9 (42)	7 (41)	9 (53)
Energy intake (kcal) (mean, SD)	1,899 (636)	2,035 (690)	2,127 (679)	1,623 (564)	2,446 (719)	1,498 (547)	2,434 (717)

^i,ii^The letters indicated significantly different values (*p* < 0.001 and *p* < 0.0001 respectively) performed by CMH test for homogeneity over all quintiles.

### 3.2. Nutrient adequacy and dietary diversity

Generally, MPA was low in the total population (0.38, Table 3). The Omnivorous and Pescatarian patterns had similar MPA values (0.51 for both in Q5 (highest adherence) and 0.30 and 0.24 in Q1 (lowest adherence), respectively). For the Traditional pattern, the MPA for Q5 was slightly lower than the other patterns (0.45). In terms of dietary diversity, mean DDS across the total population was low and did not meet the 5-point cut off proposed by FAO (43); in fact, only 42.8% of the total population achieved minimum dietary diversity (Table 3). When considering the individual dietary patterns, both the highest and the lowest DDS were found in the Omnivorous pattern (71.6% for Q5 and 14.7% for Q1). DDS for Q5 and Q1 were comparable in the Pescatarian and Traditional patterns (54.2 and 56.5%; and 32.7 and 27.8%, respectively). Looking at diet quality as a whole, the Omnivorous pattern performed best, as shown by the highest MPA (0.51 ± 0.21) and DDS (5.4 ± 1.3).

**Table 3:** The mean probability of adequacy (MPA)^a^ and the dietary diversity score (DDS) in the total population and in Q1 and Q5 of 3 dietary patterns of household (per AFE) in the Vietnam General nutrition survey 2009–2010 dataset.

	Total population	Omnivorous pattern		Traditional pattern		Pescatarian pattern	
		Q1	Q5	Q1	Q5	Q1	Q5
	*n* = 8,225	*n* = 1,645	*n* = 1,645	*n* = 1,645	*n* = 1,645	*n* = 1,645	*n* = 1,645
MPA	0.38 (0.23)	0.30 (0.19)	0.51 (0.21) ^i^	0.33 (0.24)	0.45 (0.18)^i^	0.24 (0.22)	0.51 (0.19)^i^
DDS^b^	4.4 (1.3)	3.5 (1.0)	5.4 (1.3)^i^	3.9 (1.2)	4.8 (1.3)^i^	4.0 (1.3)	4.8 (1.3)^i^
DDS ≥ 5 (%)	42.8	14.7	71.6	27.8	56.5	32.7	54.2

^a^MPA: Mean probability of nutrient adequacy was an average of the PAs. The mean of PAs was calculated as an average of the PA of each individual and was standardized by 2,000 kcal (Supplementary Table S2).

^b^Dietary diversity score (DDS) was created based on the indicator Minimum Dietary Diversity for Women (MDD-W). The DDS was reported with the amount of consumption was greater than 15 grams of each food group.

^i^The letter indicates significantly different values (*p* < 0.001) as calculated by the linear regression of environmental impact regressors (MPA and DDS) over the quintiles of each dietary pattern.

### 3.3. Environmental impact

Table 4 presents the average GHGE and blue water use per AFE. The GHGE was 4.51 kg CO_2_-eq. and the blue water use was 0.12 m^3^ in the total population. Note that when standardizing for 2,000 kcal of energy intake, the GHGE, and blue water use increased, as mean energy intake was lower than 2,000 kcal in this population (1,899 kcal).

**Table 4:** Environmental footprints in total population and in Q1 and Q5 of 3 dietary patterns of household (per AFE) in the Vietnam General nutrition survey 2009–2010 dataset.

	Total population	Omnivorous pattern		Traditional pattern		Pescatarian pattern	
		Q1	Q5	Q1	Q5	Q1	Q5
	*n* = 8,225	*n* = 1,645	*n* = 1,645	*n* = 1,645	*n* = 1,645	*n* = 1,645	*n* = 1,645
GHGE (kgCO_2_eq)	4.51 (2.54)	3.92 (1.48)	6.29 (3.58)^i^	4.10 (2.24)	5.44(2.53)^i^	3.61(2.67)	5.85 (2.74)^i^
GHGE per 2,000 kcal	4.82 (2.49)	3.87 (0.69)	6.14 (3.57)^i^	5.12 (2.47)	4.48(1.81)^i^	4.94 (3.40)	4.88 (2.09)^i^
Blue water (m^3^/kg)	0.12 (0.05)	0.12 (0.04)	0.15 (0.04)^i^	0.10 (0.04)	0.16(0.05)^i^	0.10 (0.04)	0.15 (0.05)^i^
Blue water per 2,000 kcal	0.13 (0.02)	0.11 (0.01)	0.14 (0.04)^i^	0.12 (0.03)	0.13 (0.02)^i^	0.13 (0.03)	0.12 (0.02)^i^

^i^The letter indicates significantly different values (*p* < 0.001) as calculated by the linear regression of environmental impact regressors (GHGE and blue water use) over the quintiles of each dietary pattern.

For the dietary patterns, adherence to the Omnivorous pattern was positively associated with both total GHGE and GHGE per 2,000 kcal. For the Traditional and Pescatarian patterns, adherence was also positively associated with total GHGE, but an inverse association was found when standardizing GHGE per 2,000 kcal. That is, GHGE per 2,000 kcal decreased from Q1 to Q5 in these two patterns. For blue water use, a positive association with higher adherence was found for all three patterns, however for the Pescatarian pattern this association was reversed when blue water use was expressed per 2,000 kcal.

**Figure 1:**
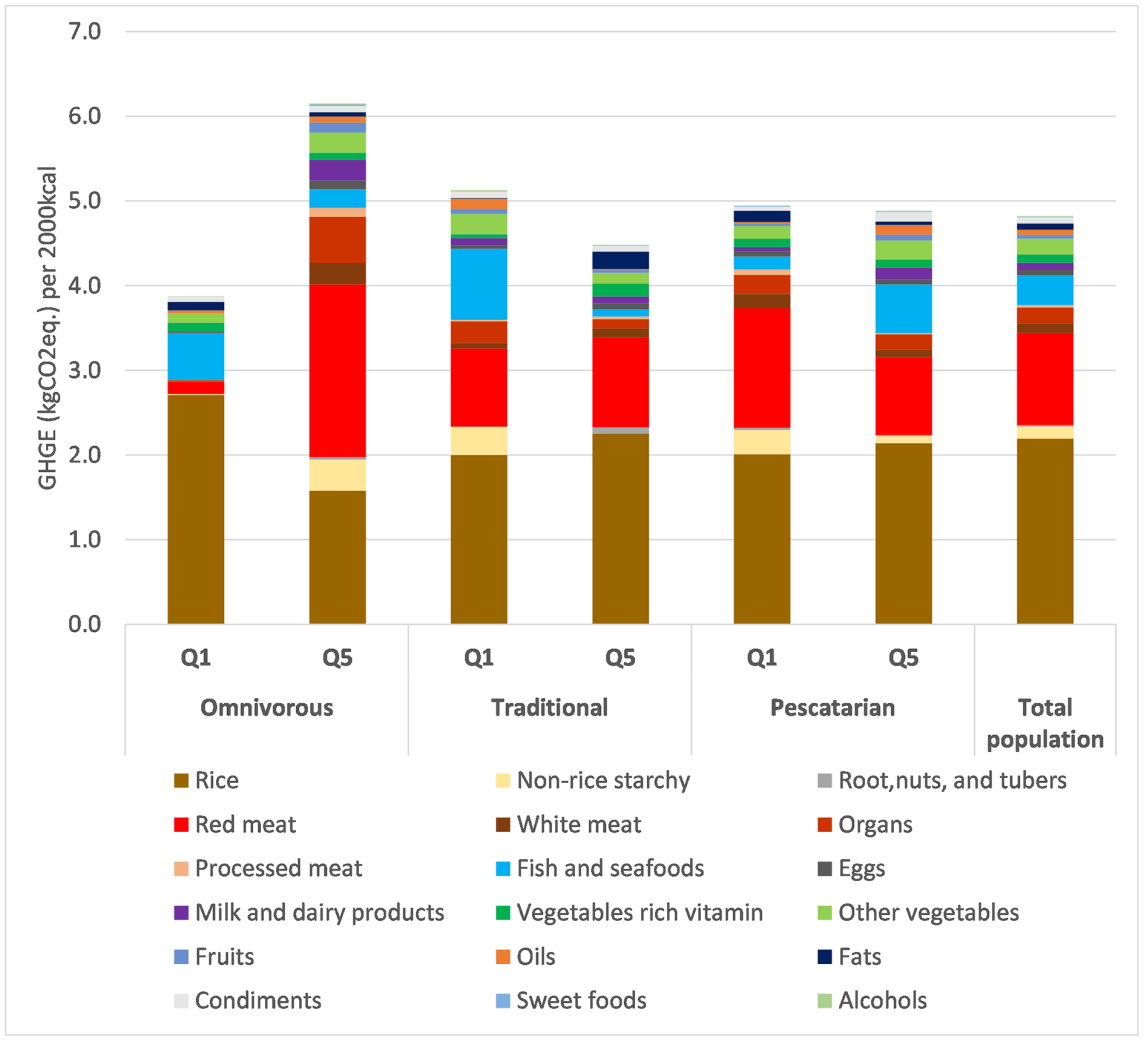
GHGE per 2,000 kcal for Q1 and Q5 of 3 dietary patterns for household (per AFE) in the Vietnam General nutrition survey 2009–2010 dataset. *n* = 1,645 per each quintile.

Figure 1 describes the GHGE per 2,000 kcal for the three patterns and total population. The GHGE of Q5 of the Omnivorous pattern was the highest due to the large contribution of red meat and other meat food groups, which was higher than Q1 and all quintiles in both other dietary patterns. In the Traditional pattern, despite a higher GHGE from red meat and rice in Q5 compared to Q1, the total GHGE per 2,000 kcal in Q5 was lower than in Q1 due to lower GHGE from fish and other seafoods. Regarding the Pescatarian pattern, the total GHGE per 2,000 kcal of Q5 and Q1 were comparable, as a larger contribution of fish and seafoods in Q5 was compensated by a lower contribution of meat.

**Figure 2:**
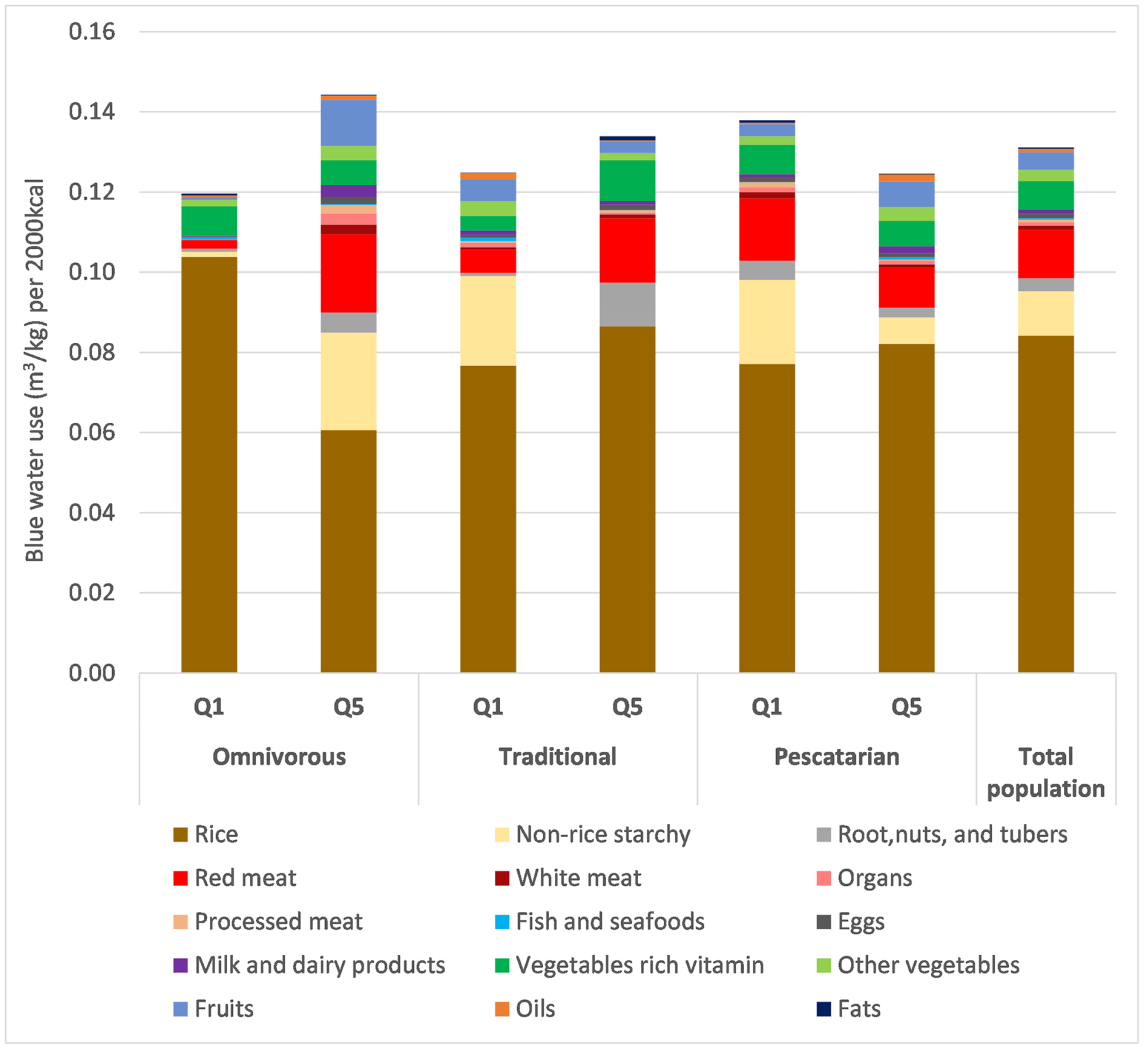
Blue Water use per 2,000 kcal for Q1 and Q5 of 3 dietary patterns for household (per AFE) Vietnam General nutrition survey 2009–2010 dataset. *n* = 1,645 per each quintile.

Figure 2 shows the contribution of blue water use by food group across the three patterns and total population. Rice was responsible for the largest contribution of blue water use in all quintiles across all patterns. The water use contribution from red meat was higher in Q5 than in Q1 for both Omnivorous and Traditional patterns, whereas a reverse trend was observed in the Pescatarian pattern. Finally, the Omnivorous pattern was responsible for both the highest blue water use (Q5) and the lowest blue water use (Q1) across all quintiles in all patterns.

## 4. Discussion

We identified three distinct dietary patterns: an Omnivorous pattern with a high consumption of red meat, a rice-based pattern with characteristics of the traditional Vietnamese diets (Traditional pattern), and a Pescatarian pattern in which fish was mainly consumed. The Omnivorous pattern was predominantly consumed in the plateaus and by the wealthiest strata of the population, while the Traditional pattern was more often found in the mountainous areas and in populations with the lowest wealth. The Pescatarian pattern was more prevalent in the coastal region, while its association with wealth was weaker. Our results show that diet quality in terms of nutrient adequacy and diet diversity of Vietnamese women was low and varied according to adherence to the dietary patterns. The environmental impact of diets was higher than the 2050 target according to the work of de Pee et al. (49) as adapted from Willett et al. (2). Across the total population, estimated GHGE per 2,000 kcal was 4.82 kg CO_2_-eq. per 2,000 kcal and blue water use was 0.12 m^3^ per 2,000 kcal and was highest in the Omnivorous pattern.

While the richer populations adhered more often to the Omnivorous pattern, the poorer populations adhered most to the Traditional pattern. The transition from a rice-based diet to a diet higher in consumption of red meat and sweet foods in the Omnivorous pattern reflects Westernization of the dietary intake in the richer populations of the delta regions. A similar so-called nutritional transition pattern and a staple diet pattern (comparable to the Omnivorous and Traditional patterns, respectively, identified in this study) were found in studies in Central Java, Indonesia, and northeast Thailand (50, 51).

In the present study, the average diets of Vietnamese women showed a low diversity (mean DDS was 4.4) and a high risk of micronutrient inadequacy (mean MPA was 38%). The mean DDS was lower than observed in China (5.0) (52). As for the MPA in the total population, this was higher than results from studies conducted in Bangladesh (35%) and Philippines (34%) in 2011 (47) and in China (30%) using the same set of nutrients (52). The low MPAs in both the total population and across all the dietary patterns reflect the high prevalence of micronutrient deficiency in Vietnam. According to National Institute of Nutrition, in 2015, on average, the prevalence of anemia was 25.5%, iron deficiency was 37.7%, and zinc deficiency was 63.6% in Vietnamese women (53).

To limit the environmental impact of various diets, global recommendations for GHGE emissions exist, such as provided by the EAT-Lancet which suggests a target of 1.36 kg CO_2_-eq. (2, 49). The findings of our study showed that the average diet of the total population (4.82 kg CO_2_-eq.) exceeded this recommendation. The mean GHGE of the Vietnamese diet was also higher compared to other studies in Vietnam, such as those of Heller et al. (approximately 3.33 CO_2_-eq. per person) and Trinh et al. (3.17 kg CO_2_-eq. per person) (25, 26). These differences may be explained by the use of different methods used in calculations. While we applied Clune’s dataset (54), which considered the full value chain from farm (production) to fork (consumption), the dataset of Heller et al. (which was also used by Trinh et al.) calculated the GHGE of food to the farm gate level, leaving transportation and consumption unaccounted for. The GHGE in our study was also higher than those found in China (2.9 kg CO_2_-eq. per person) using a dataset from the same period, but lower than those reported for certain other regions with comparable diets, such as Hong Kong (5.7 kg CO_2_-eq. per person per day) (55). The GHGE from our Vietnamese study were also lower than those of high income countries, such as the Netherlands, Chile, and Japan (56, 57).

There were significant differences of environmental impacts between Q1 and Q5 across the three dietary patterns. The Omnivorous pattern was characterized by a high intake of red meat, explaining the high GHGE of this pattern compared to the other two patterns. This corroborates with the finding of Rosi et al., in that the Omnivorous pattern in their study had a daily carbon emission 1.5x higher (4.0 kg CO_2_-eq. per day) than the ovo-lacto-vegetarian diet (2.5 kg CO_2_-eq.) (58). Blue water use also significantly differed between the patterns and the quintiles. For both the Omnivorous and Pescatarian patterns significant differences existed between Q1 and Q5, which can be owed to differential intake of rice and fruits, two food groups that require larger quantities of blue water to be cultivated. These findings, too, align with existing results, such as those of Kim et al. studying the contribution of plant foods and aquatic animals in the Pescatarian diet in India and Indonesia (57).

At the level of specific foods or food groups differences existed, too. Rice also contributed the most to the GHGE and blue water use in two of three dietary patterns (Traditional and Pescatarian pattern). In our study, the starchy staples, especially rice, contributed the most to the blue water footprint. The findings on blue water footprint were similar to a study in Vietnam from Trinh et al. but lower than a study from China (1.8 m^3^) (26, 55).

Overall, neither of the recommendations for health or those for environmental impact set by the EAT-Lancet were met by any of the three derived dietary patterns at the highest level of adherence (Q5). Even the Omnivorous pattern, which performed best from the perspective of diet quality, was still far from the EAT-Lancet recommendations (2, 49), and had the highest GHGE of all the patterns. Although GHGE was lower in the Traditional pattern, diet quality was the lowest of all the patterns. The Pescatarian pattern showed a marginally lower DDS than the omnivorous and an equivalent MPA; but in terms of environmental impact, the Pescatarian pattern performed better than the Omnivorous pattern but not as well as the Traditional pattern.

The findings of dietary patterns and their distributions within the regions suggest a role for targeted resource allocation in policy and government in improving diet quality and reducing environmental impact of diet by using local food-based strategies to be suitable for food and social norms in each region. This could be manifested by the implementation strategies on communication such as nutritional counseling and healthy diet advertisement campaigns in the urban and suburban areas, where consumption of meat and sweet foods were higher. In contrast, in the mountainous and poor communities, policies to improve the micronutrient status and to increase dietary diversity should be prioritized by for example promoting or facilitating home gardening, small animal husbandry, poultry and social marketing (59). In addition, the GHGEs we found were higher than recommended (EAT-Lancet commission) but were still lower than other high-income countries. National policies, including the National Nutrition Strategy 2010–2020, have identified necessary measures to improve the health status of the Vietnamese, however failed to consider the environmental impact of the diet and the importance of regional differences in dietary patterns. Results of the present study may help to guide a targeted approach to improve toward healthy and environmentally sustainable diets for everyone, everywhere in Vietnam. Thus, major changes in food systems in Vietnam will be required in order to allow for sustainable diets that are both healthier and reduce environmental impact.

Several limitations of this paper are acknowledged. Firstly, there were shortcomings in the dataset. The intake data used in the study was from 2010 and might not be representative of the current overall food intake. Nevertheless, the GNS was selected due to its availability, a large sample size and because of the nationally and regionally representative nature, meaning mean values are more likely to be accurate and outliers are better detected. Moreover, these advantages of the GNS provide a more complete picture of diet quality on the national and regional levels, although the aforementioned shortcomings should be considered. Secondly, at the time of data collection, the method of collection of food intake data was a single 24 h recall and at the household level, which is unable to estimate the usual intake of the individual. Despite the AFE method was validated and used to convert the data from household-level food consumption into individual-level intakes, the intake of protein and iron may be overestimated in adult women if the intra-household allocation of these nutrients was not equitable (31). The AFE method is based on the assumption that food is distributed in the household based on energy requirement. However, we do acknowledge this often is not the case, and distribution of food is often according to status in the household (31), meaning that the distribution for women is not equitable (or lower) than expected when based on their energy requirements. Furthermore, due to the limitation of this method, this study could not examine the individual characteristics (educational level, occupations) of these derived dietary patterns. Thirdly, at the time of this research, there were no Vietnam-specific GHGE or water footprint datasets available, therefore we selected the next most appropriate existing dataset to generate the environmental imprints. The environmental impact datasets were based on global estimations which will likely differ from the actual situation of Vietnam. By using external GHGE and BWU datasets, it may thus not reflect the true values of GHGE and BWU of Vietnamese foods. Nevertheless, applying the GHGE value at the food items level minimizes the under- or overestimation compared to only calculating at the food group level. Finally, the environmental impacts of food are not only limited to GHGE and BWU; land use, eutrophication, acidification, and food waste also have environmental consequences. However, limited availability to data on these variables prevented their incorporation into the current study.

## 5. Conclusion

To the best of our knowledge, this paper is the first to use national intake data to characterize the differences in environmental impacts from derived dietary patterns—Omnivorous, Traditional and Pescatarian- of Vietnamese women. All dietary patterns have higher but still low nutrient adequacy while, when standardized, showing similar environmental impacts compared to the total population. This indicates that there is need to optimize existing diets for nutrient adequacy and environmental impact. As the distribution of the patterns in the regions and in socio-economic groups differ, strategies to optimize dietary intake should be differentiated between region or income groups. Given the desirability of achieving diets that fulfill the criteria of being both healthy and environmentally sustainable, deliberation on solutions to this issue is warranted.

## Data availability statement

The datasets presented in this article are not readily available because the dataset is the property of the National Institute of Nutrition (NIN) in Vietnam, and we obtained permission to use it as part of the collaborative effort between NIN and Wageningen University and Research. However, we are not authorized to distribute the dataset to any third party. Requests to access the datasets should be directed to ninvietnam@viendinhduong.vn.

## Ethics statement

This study was reviewed and approved by the ethics committee of the National Institute of Nutrition, Vietnam. All participants provided informed consent prior to participation in this study.

## Author contributions

SN, ET, and IB developed the idea of the manuscript. SN developed the analysis plan of the study, conducted the statistical analysis, and wrote the manuscript. SB, ET, IB, EF, and TL contributed to developing the methods, interpreting the results, and improving the writing of the manuscript. All authors reviewed and approved the final version submitted for publication.

## Funding

This work was undertaken as part of the CGIAR Research Program on Agriculture for Nutrition and Health (A4NH; https://a4nh.cgiar.org/our-research/flagship-1/), and the CGIAR Initiative Sustainable Healthy Diets through Food system Transformation (SHiFT) (Sustainable Healthy Diets - CGIAR; https://www.cgiar.org/initiative/sustainable-healthy-diets/).

## Conflict of interest

The authors declare that the research was conducted in the absence of any commercial or financial relationships that could be construed as a potential conflict of interest.

## Publisher’s note

All claims expressed in this article are solely those of the authors and do not necessarily represent those of their affiliated organizations, or those of the publisher, the editors and the reviewers. Any product that may be evaluated in this article, or claim that may be made by its manufacturer, is not guaranteed or endorsed by the publisher.
