# Supplementary material for: Environmental impact and nutrient adequacy of derived dietary patterns in Vietnam
Source: Front Nutr. 2023 Jul 6;10:986241. doi: 10.3389/fnut.2023.986241 (PMC10358330; doi:10.3389/fnut.2023.986241)
Supplement: Supplementary file 1 [file Data_Sheet_1.docx]

Supplementary Material

Environmental Impact and Nutrient Adequacy of Derived Dietary Patterns in Vietnam.

**Son D Nguyen^1,2*^, Sander Biesbroek^1^, Tuyen D Le^2^,**

**Edith J.M. Feskens^1^, Inge D Brouwer^1^, Elise F Talsma^1^**

^1^ Division of Human Nutrition and Health, Wageningen University, P.O. Box 17, 6700 AA Wageningen, The Netherlands.

^2^ National Institute of Nutrition, Ministry of Health, Hanoi, Vietnam.

***Correspondence:**

Son D Nguyen

son.nguyen@wur.nl

# Supplementary Tables

**Table S1.** Food group selection for principal component analysis.

|  | **Food group from the Vietnamese Food Pyramid** | **Environmental consideration of disaggregating food group** | **Reason** | **Final selection of food group** |
| --- | --- | --- | --- | --- |
| 1 | Grains (rice, corn,wheat…) | - 1. Rice   2. Other starchy   3. Tubers | - Differences of cultivation methods of rice and other starchy can lead to different impact of water footprint and GHG emissions.  -Quantity of consumption | 1. Rice 2. Other Starchy (corn, wheat…) 3. Root and tubers, nuts, and seeds |
| 2 | Vegetables | 2. Vegetables |  | 1. Rich vitamin vegetables 2. Other vegetables |
| 3 | Fruits | 3. Fruit |  | 1. Fruits |
| 4 | Protein (Animal-based and plant-based sources) | 4.1 White meat (chicken, duck….)  4.2 Red meat (beef, pork, lamb, organ)  4.3 Fish  4.4 Seafood  4.5 Eggs  4.6 Pulses  4.7 Nuts and seeds | Beef, lambs and prawns (farmed) are top 3 GHG emission per 100 grams of protein (1).  Red meat and processed meats also have negative health impacts(2,3) | 1. White meat 2. Red meat 3. Processed meats 4. Organs 5. Fish and Seafood 6. Eggs |
|  |  |  |  |  |
|  |  |  |  |  |
|  |  |  |  |  |
| 5 | Milk | 5.1 Milk and dairy products | Milk contributes 9.5 kg CO_2_eq per 100 grams of protein while cheese produce 10.82 kg CO_2_eq per 100grams of protein(1). | 1. Milk and dairy product |
| 6 | Oil, fat | 6.1 Oil and fat |  | 1. Oil 2. Fat |
| 7 | Salt (salt, condiments…) |  |  | 1. Condiments |
| 8 | Sugar (cookies, cakes, sweet beverage, condescend milk) | 8.1 Sugar, cookies, cakes  8.2 Coffee and tea  8.3 Sweet beverages (fruit juices, sodas, sweet soups, etc.)  8.4 Liquor, wine, beers |  | 1. Sweet foods (included sweet beverages) 2. Liquor and alcohol |

**Table S2**: The probability of adequacy (PA) and mean probability of adequacy (MPA) of nutrients in the total population and in Q1 and Q5 of 3 dietary patterns of household (per AFE) in the Vietnam General nutrition survey 2009-2010 dataset.

| Nutrient | Total population | Omnivorous pattern | | Traditional dietary pattern | | Pescatarian pattern | |
| --- | --- | --- | --- | --- | --- | --- | --- |
|  |  | Q1 | Q5 | Q1 | Q5 | Q1 | Q5 |
|  | N=8225 | N=1645 | N=1645 | N=1645 | N=1645 | N=1645 | N=1645 |
| Protein (g) | 0.8 (0.4) | 0.1 (0.27) | 0.9 (0.3) | 0.8 (0.5) | 0.80 (0.3) | 0.60 (0.5) | 0.80 (0.2) |
| Vitamin C (mg) | 0.21 (0.38) | 0.34 (0.49) | 0.42 (0.5) | 0.17 (0.43) | 0.27 (0.38) | 0.12 (0.45) | 0.33 (0.4) |
| Vitamin B12 (mg) | 0.38 (0.46) | 0.63 (0.36) | 0.53 (0.51) | 0.60 (0.63) | 0.28 (0.39) | 0.24 (0.51) | 0.57 (0.43) |
| Zinc (mg) | 0.67 (0.4) | 0.32 (0.32) | 0.84 (0.33) | 0.55 (0.47) | 0.76 (0.24) | 0.45 (0.47) | 0.78 (0.24) |
| Calcium (mg) | 0.31 (0.31) | 0.31 (0.42) | 0.39 (0.31) | 0.30 (0.38) | 0.38 (0.22) | 0.15 (0.29) | 0.46 (0.26) |
| Vitamin E (mg) | 0.47 (0.47) | 0.35 (0.38) | 0.64 (0.5) | 0.43 (0.55) | 0.63 (0.41) | 0.40 (0.63) | 0.63 (0.39) |
| Vitamin B6 (mg) | 0.47 (0.44) | 0.14 (0.31) | 0.67 (0.39) | 0.32 (0.43) | 0.61 (0.32) | 0.23 (0.39) | 0.67 (0.31) |
| Folate (ug) | 0.20 (0.36) | 0.20 (0.33) | 0.29 (0.42) | 0.08 (0.26) | 0.38 (0.41) | 0.13 (0.41) | 0.28 (0.37) |
| Magnesium (mg) | 0.30 (0.41) | 0.02 (0.09) | 0.46 (0.42) | 0.25 (0.42) | 0.35 (0.36) | 0.14 (0.36) | 0.52 (0.37) |
| Iron (mg) | 0.03 (0.13) | 0.28 (0.4) | 0.07 (0.15) | 0.02 (0.11) | 0.04 (0.09) | 0.02 (0.12) | 0.06 (0.13) |
| Vitamin A (ug) | 0.38 (0.43) | 0.1 (0.27) | 0.53 (0.48) | 0.23 (0.44) | 0.56 (0.41) | 0.32 (0.57) | 0.44 (0.41) |
| MPA | 0.38 (0.23) | 0.30 (0.19) | 0.51 (0.21) i | 0.33 (0.24) | 0.45 (0.18) i | 0.24 (0.22) | 0.51 (0.19) |
